# Optimization and partial characterization of culture conditions for the production of alkaline protease from *Bacillus licheniformis* P003

**DOI:** 10.1186/2193-1801-2-506

**Published:** 2013-10-04

**Authors:** Palash Kumar Sarker, Saimon Ahmad Talukdar, Promita Deb, SM Abu Sayem, Kaniz Mohsina

**Affiliations:** Microbial Biotechnology Division, National Institute of Biotechnology, Savar, Dhaka, Bangladesh; Department of Genetic Engineering and Biotechnology, Shahjalal University of Science and Technology, Sylhet, Bangladesh

**Keywords:** Bacillus licheniformis, Alkaline protease, Shake flask culture, Production optimization

## Abstract

Proteolytic enzymes have occupied a pivotal position for their practical applications. The present study was carried out under shake flask conditions for the production of alkaline protease from *Bacillus licheniformis* P003 in basal medium containing glucose, peptone, K_2_HPO_4_, MgSO_4_ and Na_2_CO_3_ at pH 10. The effect of culture conditions and medium components for maximum production of alkaline protease was investigated using one factor constant at a time method along with its characterization. Maximum level of enzyme production was obtained after 48h of incubation with 2% inoculum size at 42°C, under continuous agitation at 150 rpm, in growth medium of pH 9. Highest enzyme production was obtained using 1% rice flour as carbon source and 0.8% beef extract as organic nitrogen source. Results indicated that single organic nitrogen source alone was more suitable than using in combinations and there was no significant positive effect of adding inorganic nitrogen sources in basal medium. After optimization of the parameters, enzyme production was increased about 20 fold than that of in basal medium. The crude enzyme was highly active at pH 10 and stable from pH 7–11. The enzyme showed highest activity (100%) at 50°C, and retained 78% relative activity at 70°C. Stability studies showed that the enzyme retained 75% of its initial activity after heating at 60°C for 1h. The enzyme retained about 66% and 46% of its initial activity after 28 days of storage at 4°C and room temperature (25°C) respectively. Mn^2+^ and Mg^2+^ increased the residual activity of the enzyme, whereas Fe^2+^ moderately inhibited its residual activity. When pre-incubated with Tween-20, Tween-80, SDS and H_2_O_2_, each at 0.5% concentration, the enzyme showed increased residual activity. These characteristics may make the enzyme suitable for several industrial applications, especially in leather industries.

## Background

Proteases execute a large variety of functions and have numerous applications in detergent, food, pharmaceutical and leather industries (Gupta et al. 2002). Alkaline proteases hold a major share of the enzyme market with two third shares in detergent industry alone (Anwar and Saleemuddin 2000, Haki and Rakshit 2003). Although there are many microbial sources available for protease production, only a few are considered as commercial producers (Beg et al. 2002). Of these, species of *Bacillus* dominate in the industry (Gupta et al. 2002). Due to enhancement of such demand of proteases for specific properties, scientists are looking for newer sources of proteases. In addition, for effective use in industries, alkaline proteases need to be stable and active at high temperature and pH and in the presence of surfactants, oxidizing agents, and organic solvents (Johnvesly and Naik 2001, Fu et al. 2003, Rahman et al. 2006, Bhunia et al. 2011). Culture condition is also another important parameter to consider that can influence the cost and rate of enzyme production (Beg et al. 2002). About 30-40% of the cost of industrial enzymes depends on the cost of the growth medium (Joo et al. 2003). In addition, extracellular protease production from *Bacillus* species is significantly influenced by medium composition and some physical factors, such as fermentation period, aeration, inoculum density, incubation temperature and pH of growth medium (Puri et al*.*^2002^; Genckal and Tari 2006; Nadeem et al. 2006). In this study, strategies were applied to optimize the production of alkaline protease from *Bacillus licheniforms* P003. The enzyme was also partially characterized in this study.

## Results and discussion

The demand of eco-friendly technology is increasing day by day to reduce the pollution at industrial level. Being an eco-friendly compound, enzymes got application at different industrial sectors. There is no general medium for protease production by different microbial strains (Pandey et al. 2000). Every microorganism evidences its own idiosyncratic physicochemical and nutritional requirements for growth and enzyme secretion (Reddy et al. 2008). Enzyme production was carried out under shake flask culture at 37°C and 150 rpm for the production of alkaline protease from *B. licheniformis* P003 in basal medium containing Glucose 10.0 g/l, Peptone 10.0 g/l, K2HPO4 1.0 g/l, MgSO4 0.2 g/l, Na2CO3 5.0 g/l at initial pH 10. After 48 h fermentation, enzyme activity of 46.10 U/ml was obtained. To increase the production of enzyme, optimization of culture conditions and medium components were then studied. Initially, the traditional one-fact-at-a-time method was employed for selection of appropriate medium ingredients and their apparent concentrations of the enzyme production.

The initial pH of the culture media has an intense effect on protease production. It was found that *B. licheniformis* P003 was capable of producing alkaline protease over a wide range of initial culture pH (8.5-10.5), but the enzyme production was found to be highest (89.30 U/ml) at pH 9.0. The enzyme production was drastically reduced at pH 12.0 (Table 1). In order to assess the effect of temperature on protease production, fermentation was carried out at different temperatures ranges from 32°C to 45°C. The effect of temperature on alkaline protease production revealed that maximum yield (54.12 U/ml) was obtained at 42°C after 48 h of incubation. The maximum extracellular soluble protein was also found at 42°C. Appreciable amount of enzyme production occurred at temperatures ranging from 37 to 42°C. A decrease in enzyme yield was observed with further increase in temperature (Table 1).

**Table 1:** **Effect of culture conditions for production of extracellular protease from** ***B. licheniformis*** **P003 in shake-flask cultivation**

Culture condition	Protease activity (U/ml)	Relative activity (%)	Protease activity (U/mg)	Total soluble protein (mg/ml)
	Mean±SE		Mean±SE	Mean±SE
Initial pH				
8.0	39.01±0.190	43.68	43.72±1.22	0.889±0.021
8.5	81.28±0.199	91.02	86.13±2.12	0.944±0.021
9.0	89.30±0.661	100.00	104.10±1.53	0.858±0.016
9.5	79.38±0.143	88.89	83.81±1.51	0.947±0.016
10.0	80.55±0.194	90..20	94.41±1.45	0.853±0.015
10.5	74.62±0.562	83.56	90.70±5.48	0.825±0.057
11.0	10.91±0.185	12.22	14.74±0.09	0.74±0.010
Incubation temperature (°C)				
32	39.08±0.096	72.21	35.33±0.11	1.106±0.006
35	45.10±0.070	83.33	40.60±0.40	1.112±0.013
37	48.71±0.026	90.00	42.42±0.22	1.143±0.014
40	50.19±0.053	92.74	43.16±0.49	1.155±0.023
42	54.12±0.056	100.00	43.10±0.27	1.255±0.007
45	43.90±0.098	81.12	35.17±0.20	1.247±0.010
Incubation period (hr)				
24	9.62±0.062	20.00	7.80±0.07	1.231±0.008
48	48.10±0.062	100.00	40.74±0.17	1.178±0.008
72	36.68±0.046	76.26	30.51±0.12	1.200±0.007
96	34.27±0.072	71.25	29.92±0.11	1.143±0.007
120	22.85±0.046	47.51	20.00±0.04	1.141±0.005
Inoculums volume (%, ml)				
0.5	13.89±0.026	25.95	16.31±0.09	0.849±0.008
1.0	35.48±0.026	66.29	41.51±0.20	0.853±0.006
1.5	36.68±0.053	68.54	43.12±0.17	0.848±0.008
2.0	53.52±0.030	100.00	58.54±0.37	0.915±0.006
2.5	19.24±0.036	35.95	27.15±0.38	0.709±0.009
3.0	13.23±0.026	24.72	19.23±0.17	0.689±0.008
3.5	12.38±0.035	53.03	23.31±0.39	0.531±0.010

Data represent as mean ± standard error (SE) for three replicates.

*B. licheniformis* P003 was cultivated in basal medium at pH 10.0 for different incubation period ranging from 24 to 120 hours at a temperature of 37°C and 150 rpm and enzyme assay was carried out every 24 hours interval. The time course data revealed that maximum level of alkaline protease was produced (48.10 U/ml enzyme activity and 1.178 mg/ml protein concentration) after 48h of cultivation period (Table 1). Xiong et al. (2010) found maximum protease production by *B. licheniformis* after 48 h of incubation. Olajuyigbe and Ajele (2008) also found maximum protease production of 18.4 U/ml after 48 h incubation by *B. licheniformis* LBBL-11. Whereas, Akcan (2012) found highest alkaline protease production after 24 h by *B. licheniformis* ATCC 12759. Inoculum concentration was also studied for maximum alkaline protease production. According to data presented in Table 1, optimum concentration of inoculum for protease production was found at 2.0%. Protease production was sharply decreased at a concentration of 2.5%. Total protein content was also highest at 2.0% inoculum concentration.

**Figure 1:**
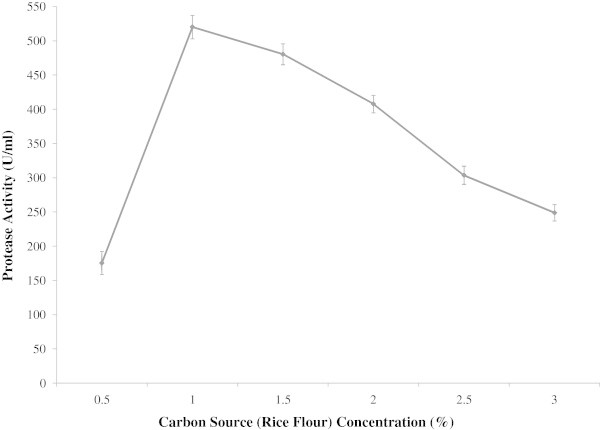
**Effect of carbon source (rice flour) concentration on protease production by** ***Bacillus licheniformis*** **P-003.** The effect of rice flour concentration on enzyme production was investigated by using 1% inoculum (w/v) in 100 ml basal medium. The fermentation was carried out at 37°C at 150 rpm for 48 h. Absorbance was measured at 660 nm with spectrophotometer and enzyme activity was presented on the y axis and carbon source concentration was on x axis. Bars represent means ± standard errors for three replicates.

To investigate the effect of different carbon sources on alkaline protease production by *B. licheniformis* P003, glucose of the basal media was replaced by equal amount of complex or simple carbon sources. It was observed that rice flour showed highest protease production followed by wheat bran and rice bran respectively (Table 2). When compared with control (52.14 U/ml), there was significant increase in enzyme yield in the case of the supplementation of rice flour (491.92 U/ml) and wheat bran (327.14 U/ml) as complex carbon sources. Similar effect of rice flour on protease production was observed by. Srividya and Mala (2011). Akcan (2012) found significant increase in enzyme yield in case of rice flour and wheat flour. In *Bacillus* strains such as *B. licheniformis* ATCC 21415 (Mabrouk et al. 1999), however, enhanced protease yields were reported on supplementation of glucose. Different concentrations of rice flour ranging from 0.5 to 3.0% were tested for maximum induction of enzyme. Maximum production occurs at 1.0% concentration and after that the production decreases as concentration increases (Figure 1).

**Table 2:** **Depicting the protease activity and total soluble protein of the culture supernatant of** ***B. licheniformis*** **P003 grown on different carbon source in presence of 1% peptone**

Carbon sources	Protease activity (U/ml)	Protease activity (U/mg)	Total protein content (mg/ml)
	Mean±SE	Mean±SE	Mean±SE
Wheat flour	120.87±0.046	78.90±0.35	1.530±0.008
Wheat bran	327.14±0.056	167.22±0.22	1.955±0.004
Rice flour	491.92±0.072	344.40±0.46	1.426±0.006
Rice bran	267.61±0.075	142.83±0.33	1.872±0.006
Corn flour	91.40±0.070	65.61±0.08	1.395±0.006
Starch	85.39±0.036	59.13±0.17	1.442±0.007
Maltose	24.05±0.082	22.65±0.08	1.059±0.008
Lactose	10.82±0.062	11.36±0.12	0.951±0.006
Fructose	55.92±0.062	62.39±0.24	0.894±0.006
Control (Glucose)	52.14±0.056	60.96±0.29	0.853±0.007

Data represent as mean ± standard error (SE) for three replicates.

The effect of different nitrogen sources in the form of organic or inorganic on alkaline protease production was also studied to identify the suitable nitrogen source, because the requirement for specific nitrogen source differs from organism to organism (Kumar and Takagi, 1999). Maximum yield (191.23 U/ml) was obtained in case of beef extract at initial concentration of 0.8% followed by yeast extract (Table 3). No significant effect was observed on protease production when combinations of different organic nitrogen sources were used at a concentration of 0.5%+0.5% (Table 3). Results indicated that single organic nitrogen source was more suitable than using them in combinations. Hossain et al. (2007) also reported that mixture of nitrogen sources were not effective to increase the production of protease by *B. licheniformis* MZK-3. These findings are in good agreement with Naidu and Devi (2005), who also reported maximum enzyme production in case of beef extract followed, by yeast extract. Whereas, Nadeem et al. (2008) reported maximum yield of enzyme in case of yeast extract followed by peptone. Nejad et al. (2009) reported that a mixture of peptone and yeast extract (0.5%, 0.5%) were the best nitrogen sources for protease production by *B. licheniformis* BBRC 100053. But, in the present study, lowest production was found when mixture of peptone and yeast extract was used in the basal medium.

**Table 3:** **Depicting the protease activity and total soluble protein of the culture supernatant of** ***B. licheniformis*** **P003 grown on different nitrogen source in presence of 1% glucose**

Nitrogen sources (%)	Protease activity (U/ml)	Protease activity (U/mg)	Total protein content (mg/ml)
Control (Peptone, 1%)	37.66±0.311	25.61±0.48	1.080±0.010
Tryptone (1%)	31.87±0.165	20.49±0.88	1.543±0.084
Yeast Extract (1%)	48.71±0.233	44.06±0.08	1.102±0.010
Beef Extract (1%)	191.23±0.229	159.37±1.31	1.190±0.026
P (0.5%) + T (0.5%)	22.85±0.145	13.67±0.67	1.673±0.075
P (0.5%) + YE (0.5%)	12.02±0.106	8.85±0.20	1.356±0.020
P (0.5%) + BE (0.5%)	19.84±0.145	18.30±0.15	1.082±0.006
T (0.5%) + YE (0.5%)	34.27±0.207	17.47±0.16	1.956±0.014
T (0.5%) + BE (0.5%)	21.64±0.229	12.70±0.16	1.700±0.009
BE (0.5%) + YE (0.5%)	36.68±0.257	30.40±0.36	1.207±0.007

P= Peptone, T= Tryptone, YE=Yeast Extract, BE= Beef Extract, Data represent as mean ± standard error (SE) for three replicates.

**Figure 2:**
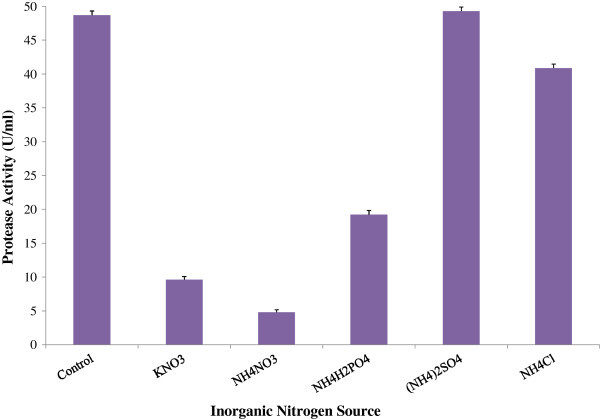
**Effect of inorganic nitrogen source on protease production by** ***Bacillus licheniformis*** **P-003.** To determine the effect of inorganic nitrogen sources on enzyme production, different inorganic nitrogen sources were used (1.0% w/v) in 100 ml of basal medium. The fermentation was carried out at 37°C at 150 rpm for 48 h. Bars represent means ± standard deviations for three replicates.

Low activity (4.81 U/ml and 9.62 U/ml) was found in case of NH_4_NO_3_ and KNO_3_ respectively while highest activity (49.31 U/ml) was found when (NH_4_)_2_SO_4_ was used as sole inorganic nitrogen source (Figure 2). When inorganic nitrogen salts were used in the basal medium as sole nitrogen source at a concentration of 1%, maximum enzyme production was obtained by (NH_4_)_2_SO_4_. However, Bhunia et al. (2010) found maximum production in case of sodium nitrate at a concentration of 0.5% followed by potassium nitrate, ammonium chloride, ammonium nitrate and ammonium sulfate respectively. Many other researchers have also reported that organic nitrogen source was better for enzyme production than inorganic (Feng et al. 2001; Joo et al. 2003).

Alkaline protease production was significantly increased about 20 fold (930.02 U/ml) using the simulated media after optimization of different parameters in comparison to the basal medium (46.10 U/ml).

**Figure 3:**
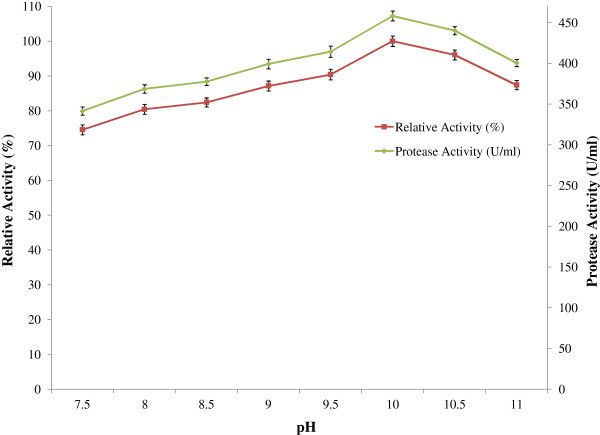
**Effect of pH on protease activity.** For determination of optimum assay pH of the enzyme reaction, 0.05M Na_2_HPO_4_-NaH_2_PO_4_ (pH 6.5 to 7.0), Tris- HCl (pH 7.5 to 8.5) and Glycine-NaOH (pH 9.0 to 12) buffers were used. The reaction was carried out for 20 min at 50°C in shaking water bath. Enzyme activity was measured and the results were presented on graph. Bars represent means ± standard deviations for three replicates.

**Figure 4:**
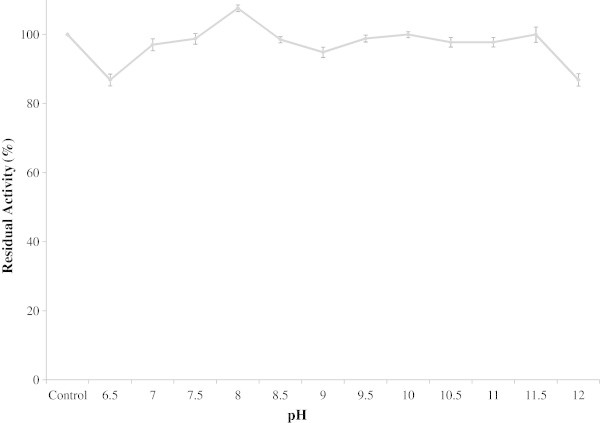
**Effect of pH stability on protease activity.** The effect of pH on the stability on protease was studied by pre-incubating the enzyme with 0.05 M buffers covering the pH range of 6.5-12.0. 0.5 ml enzyme samples were added to 1 ml of different buffer and pre-incubated at 4°C for 24 h. Then residual activity in each sample was determined by standard protease assay and compared with the control sample kept at 4°C without any buffer. Bars represent means ± standard deviations for three replicates.

The activity of the protease towards casein at different pH levels was determined. It can be observed from the figure (Figure 3) that protease was active between pH 7.5 and 11.0 and optimum at pH 10. The relative activities at pH 7.5 and 11.0 were found to be 74.54 % and 87.40% of the activity found at pH 10 respectively. The enzyme was found stable over a broad pH range 6.5 to12.0 and higher stability was observed in the range of 7.5-8.5 (Figure 4).

**Figure 5:**
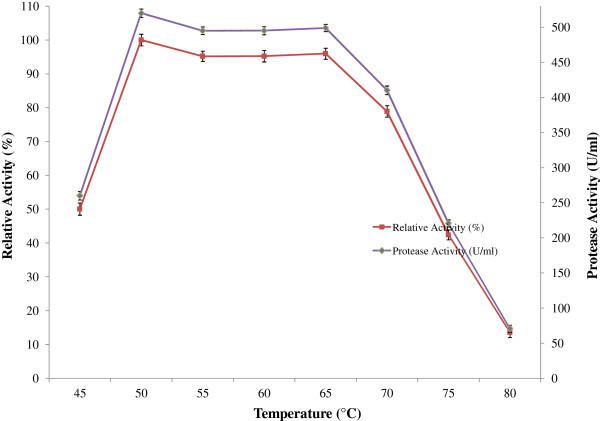
**Effect of temperature on protease activity.** To study the effect of temperature on enzyme reaction activity, enzyme reaction was carried out at different temperatures for 20 min in a shaking water bath and results are presented on graph. Bars represent means ± standard deviations for three replicates.

**Figure 6:**
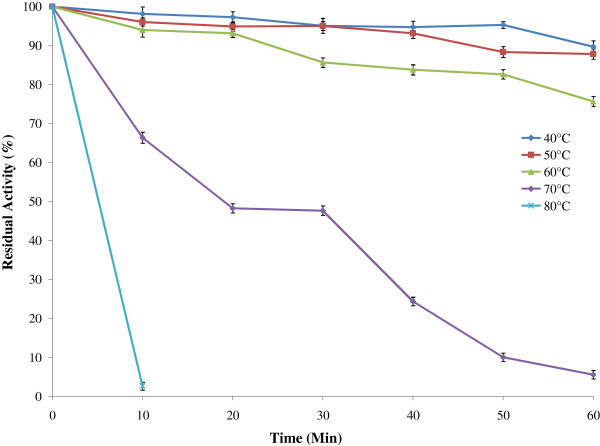
**Effect of temperature on protease stability.** For the determination of thermostability of protease, 0.5 ml of enzyme solutions were pre heated at different temperatures for different time intervals in a shaking water bath. Then enzyme activity of the heat treated enzymes were then measured and the results are presented on graph. Bars represent means ± standard deviations for three replicates.

The protease was found most active between temperature 50°C and 65°C and optimum at 50°C (Figure 5). The enzyme retained 87% and 70% of residual activity after 60 minutes of incubation at 50°C and 60°C respectively (Figure 6). Yu et al. (2006) also reported that enzyme from *B. licheniformis* MH31 was stable (75%) up to 60°C after 1 h of incubation, whereas Deng et al. (2010) found that enzyme retained about 40% of its initial activity at 60°C after 1h of incubation in case of *Bacillus sp.* B001. The most commercially available subtilisin-type protease is also active at temperature ranging from 50 to 60°C (Ghorbel et al. 2003; Saeki et al. 2007). Interestingly alkaline protease from *B. licheniformis* P003 was found to be more stable at high temperatures when compared with previous findings.

**Figure 7:**
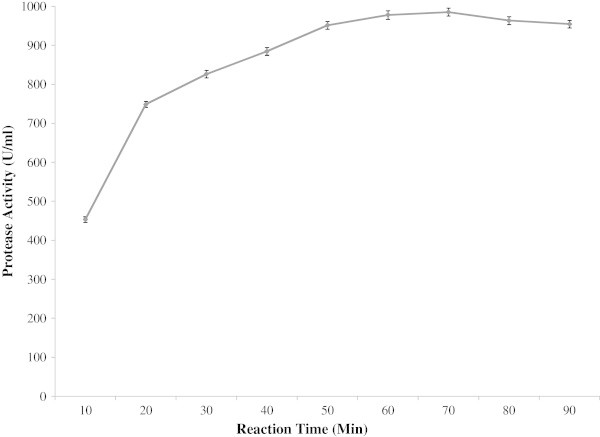
**Effect of reaction time on enzyme activity.**

**Figure 8:**
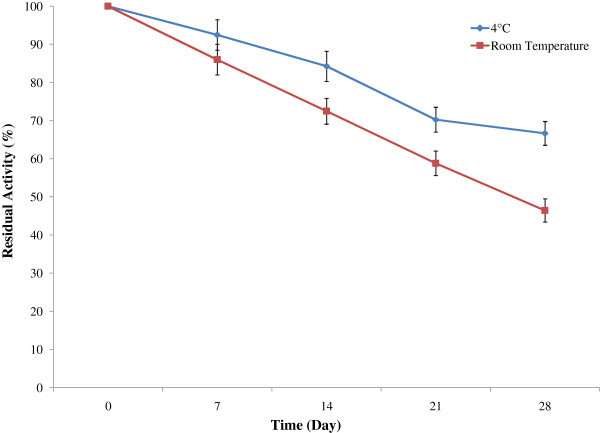
**Effect of reaction time on enzyme activity.** To investigate the optimum reaction time of the enzyme solution, reaction was carried out at 50°C in a water bath at different time intervals and the enzyme activity was then measured. The results are presented on graph. Bars represent means ± standard deviations for three replicates.

All enzymes have optimum reaction time for showing its maximum catalytic activity. The enzyme exhibited its maximum activity at 70 min of reaction time (Figure 7). Stability of the enzyme is of great importance for the economy of their industrial application since highly purified enzyme preparations are expensive. Temperature is an important limiting factor for storage of enzymes. The present enzyme showed good storage stability at 4°C (Figure 8). About 66% of the activity retained up to 28 days when stored at 4°C whereas only 46% activity retained at room temperature (30°C). When compared with control (stored at 4°C), the enzyme showed reasonable storage stability at room temperature. The results are in accordance with Paul et al. (2007).

**Figure 9:**
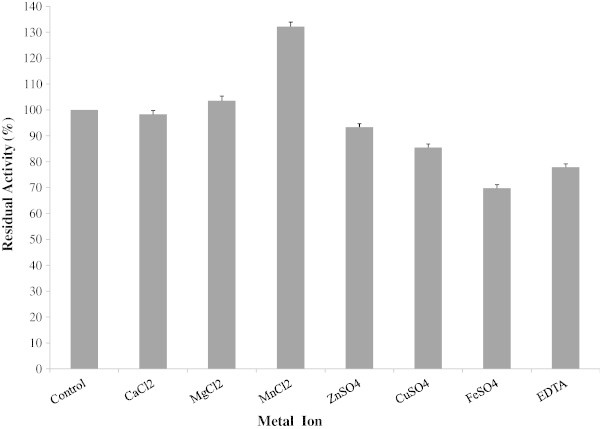
**Effect of storage temperature on stability.** To determine the storage stability of protease enzyme, crude enzyme solution was stored at 4°C and room temperature. Enzyme activity was measured at 7 days interval over 28 days by standard assay method described previously. The results are presented on graph. Bars represent means ± standard deviations for three replicates.

Different metal ions were used to determine effect of protease activity. Mn^2+^ and Mg^2+^ increased 31% and 3% of the residual activity respectively (Figure 9). Ca^2+^ had no significant effect on enzyme activity. However, Fe^2+^ and EDTA decreased the protease activity by 30.19% and 17% respectively. This result correlates with the findings of Sayem et al. (2006), who reported that Mn^2+^ and Mg^2+^ ion increased the protease activity by 39% and 31%, respectively. Yu et al. (2006) also found that Mn^2+^ and Ca^2+^ marginally stimulated the alkaline protease from *B. licheniformis* MH31 up to 20% of the maximum activity.

Studies on the effects of various surfactants and oxidizing agents at different concentrations on protease activity revealed that upon incubation with Tween-20, Tween-80 and H_2_O_2,_ the enzyme showed enhanced residual activities between 103-108%, each at 0.5% concentration (Table 4). At 1% concentration of Tween-20 and H_2_O_2_, the enzyme retained 84% and 90% residual activity. But in case of Tween-80, both 0.5% and 1% concentrations increased the protease activity up to 107% and 104%, respectively. Enzyme sharply lost its residual activity in the presence of SDS to 16% and 11% at a concentration of 0.5% and 1.0% respectively. The protease activity was decreased gradually as the concentration of Tween-20 and H_2_O_2_ increases.

**Table 4:** **Effect of surfactants and oxidants on protease stability**

Surfactants/ oxidizing agent	Concentration (%)	Activity (U/ml)	Residual activity (%)
Tween-20	0.5	497.33±0.141	103.37
	1.0	407.13±0.132	84.62
Tween-80	0.5	515.37±0.104	107.12
	1.0	503.43±0.195	104.64
SDS	0.5	78.78±0.210	16.37
	1.0	52.92±0.132	11.00
H_2_O_2_	0.5	520.79±0.195	108.25
	1.0	435.99±0.235	90.62
Control	-	481.09±0.198	100.00

Data represent as mean ± standard error (SE) for three replicates.

## Conclusion

The results in this study indicated that optimization of culture conditions played a central role for improving yield through the shake flask fermentation process. Maximum protease activity was obtained after optimization of the nutritional components and fermentation processes. Along with production optimization, this work also describes the partial characterization of alkaline protease from *B. licheniformis* P003. These characteristics indicated that alkaline protease produced by *B. licheniformis* P003 might be an excellent candidate for use as detergent additive in laundry industry and as a dehairing and bating agent in tanneries. Further studies on protease produced by *B. licheniformis* P003 would make it feasible for commercialization of the enzyme.

## Materials and methods

### Microorganism

The bacterial culture *B. licheniformis* P-003 was obtained from the Microbial Biotechnology Division, National Institute of Biotechnology, Ganakbari, Savar, Dhaka. Stock culture of the organism was maintained on nutrient agar medium at 4°C in refrigerator for routine laboratory use and 15% glycerol broth at −20°C for long term preservation.

### Preparation of seed culture

Vegetative inoculums were used in the present studies. 50 ml of inoculums medium containing nutrient broth 13 g/l, pH 7.4±0.2 was transferred to a 100 ml conical flask and cotton plugged. It was sterilized in an autoclave at 15 lbs/inch^2^ pressure at 121°C for 20 min. After cooling to room temperature, a loopful of freshly grown culture was aseptically transferred to it. The flask was incubated overnight at 37°C and 150 rpm in a rotary shaking incubator.

### Fermentation and separation of culture filtrates

The seed culture (1 ml) was transferred to 100 ml of basal medium in a 250-ml Erlenmeyer flask. Basal medium contained Glucose 10.0 g/l, Peptone 10.0 g/l, K_2_HPO_4_ 1.0 g/l, MgSO_4_ 0.2 g/l, Na_2_CO_3_ 5.0 g/l (initial pH 10.0). The inoculated flasks were placed in a thermostated orbital shaker for 48 hours, at 37°C and 150 rpm. Samples were withdrawn at regular intervals and centrifuged in a refrigerated centrifuge machine at 10000 rpm for 15 minutes at 4°C. The cell free supernatant was preserved at 4°C and used for enzyme assay and protein estimation.

### Soluble protein estimation

Extracellular soluble protein in culture filtrate was estimated by Lowry’s method using bovine serum albumin (BSA) used as Standard (Lowry et al. 1951). 2 ml of analytical reagent was added to 0.2 ml suitably diluted test samples (enzyme solution). The mixture was mixed well and allowed to stand for 10 min at 50°C. Then 0.2 ml of the folin-ciocalteau reagent was added and shaken to mix well and incubated at room temperature for about 30 min. Optical density of the reaction mixture was measured at 600 nm, against a blank prepared with 0.2 ml buffer. A standard curve was constructed with each experiment using bovine serum albumin as a known protein. The amount of the soluble protein was calculated from the standard curve as mg protein per ml of test samples.

### Determination of enzyme activity

Protease activity was determined by Anson method (Anson, 1938; Bhunia et al*.*, 2010) using 1% casein as substrate. 0.2 ml of enzyme solution was added to 0.8 ml of substrate solution (1% V/V, casein with 50 mM Glycine-NaOH buffer, pH 10.0) and incubated at 50°C for 20 min independently with respective controls. The reaction was stopped by adding 1 ml of 10% TCA followed by holding 10 min at room temperature and then subsequently followed by centrifugation at 8000 rpm for 15 min at 4°C. After that 1 ml of supernatant was added to 3ml of 0.4M Na_2_CO_3_ solution. Then 0.5 ml of Folin reagent was immediately added to each tube, vortexed and left for 30 min at room temperature. This provides coloration (measured at OD_660_ nm) equivalent to 1 μmol of tyrosine, in the presence of the Folin-Ciocalteau reagent by using a tyrosine standard curve (Folin and Ciocalteu 1927). The protease activity was expressed as the difference of absorbance at 660 nm between the control and the test sample. One unit of protease activity was defined as the amount of enzyme liberating 1 μg of tyrosine/min under assay conditions. Enzyme units were measured using tyrosine (0–100 mg) as standard.

### Optimization of fermentation parameters

The fermentation condition for protease production by *B. licheniformis* P003 was studied. The experiments were carried out systematically in such a way that the parameter optimized in one experiment was maintained at its optimum level in the subsequent experiments. Various process parameters that enhance the yield of protease under submerged fermentation were investigated by taking one factor at a time. The impact of initial pH (8.0-11.0), temperature (32-45°C), incubation time (24–120 h), inoculums concentration (0.5-3.5%) and medium composition (carbon, organic and inorganic nitrogen) were evaluated. All the experiments were conducted in triplicate and then the mean values were considered.

### Statistical analysis

Data analysis was performed using SPSS software version 10 (Chicago, USA). The results were presented as mean ± SE.
